# MiRTif: a support vector machine-based microRNA target interaction filter

**DOI:** 10.1186/1471-2105-9-S12-S4

**Published:** 2008-12-12

**Authors:** Yuchen Yang, Yu-Ping Wang, Kuo-Bin Li

**Affiliations:** 1 Institute of Molecular and Cell Biology, 61 Biopolis Drive, 138673, Singapore; 2 Center for Systems and Synthetic Biology, National Yang-Ming University, Taipei, 11221, Taiwan

## Abstract

**Background:**

MicroRNAs (miRNAs) are a set of small non-coding RNAs serving as important negative gene regulators. In animals, miRNAs turn down protein translation by binding to the 3' UTR regions of target genes with imperfect complementary pairing. The identification of microRNA targets has become one of the major challenges of miRNA research. Bioinformatics investigations on miRNA target have resulted in a number of target prediction tools. Although these tools are capable of predicting hundreds of targets for a given miRNA, many of them suffer from high false positive rates, indicating the need for a post-processing filter for the predicted targets. Once trained with experimentally validated true and false targets, machine learning methods appear to be ideal approaches to distinguish the true targets from the false ones.

**Results:**

We present a miRNA target filtering system named MiRTif (miRNA:target interaction filter). The system is a support vector machine (SVM) classifier trained with 195 positive and 38 negative miRNA:target interaction pairs, all experimentally validated. Each miRNA:target interaction pair is divided into a seed and a non-seed region. The encoded feature vector contains various *k*-gram frequencies in the seed, the non-seed and the entire regions. Informative features are selected based on their discriminating abilities. Prediction accuracies are assessed using 10-fold cross-validation experiments. Our system achieves AUC (area under the ROC curve) of 0.86, sensitivity of 83.59%, and specificity of 73.68%. More importantly, the system correctly predicts majority of the false positive miRNA:target interactions (28 out of 38). The possibility of over-fitting due to the relatively small negative sample set has also been investigated using a set of non-validated and randomly selected targets (from miRBase).

**Conclusion:**

MiRTif is designed as a post-processing filter that takes miRNA:target interactions predicted by other target prediction softwares such as TargetScanS, PicTar and miRanda as inputs, and determines how likely the given interaction is a real or a pseudo one. MiRTif can be accessed from .

## Background

MicroRNAs (miRNAs) are small RNA molecules of about 22 nucleotides that are negative regulators of protein expression. Studies have shown that these small RNAs are involved in the regulation of a variety of biological processes, including developmental timing, cell death, cell proliferation, haematopoiesis and patterning of nervous systems [1]. MiRNAs regulate gene expression at the post-transcriptional level [2,3] by two modes. In the first model, miRNAs bind to target transcripts by precise or near-precise pairing, leading to direct mRNA cleavage and degradation through a mechanism involving the RNA interference (RNAi) machinery [4,5]. In the other model, the pairing of a miRNA to its targets is usually less perfect, but still affects the mRNAs' stability. Targets are hence degraded by translational repression [6,7]. At the time of writing, miRBase [8], which is the most comprehensive miRNA repository, contains 3,963 miRNA entries from primates, rodents, birds, fish, worms, flies, plants, and viruses, among which 462 are human miRNAs (Release 8.1, May 2006). Recent work in computational prediction of miRNA targets [9-11] revealed that each human miRNA could potentially target hundreds of genes and at least 30% of the human genes could be targeted by miRNAs.

Since 2003 many computational approaches have been developed to predict miRNA targets, some of them which are publicly available [12,13]. Most of them rely on knowledge of the base pairing nature between the miRNA and the target gene in animals and plants. The general idea is to find complementarity between the 3'-UTR of the potential targets and miRNAs, with emphasis on the critical pairing at the 5' end of the miRNA [14,15], which is often called the "seed" region. The conservation of the target 3'-UTR sequences in orthologous genes, the kinetic and thermodynamics of the base pairing are also useful criteria. Approaches differ in the methods used to measure conservation and to predict single [16] or multiple [17] binding sites in miRNA targets, and in the statistical approaches chosen. Position-based features, captured by machine learning classifiers, were also introduced in recent studies to model the shape and the mechanism of the seed pairing [18-20].

In spite of its progress, computational prediction of miRNA targets is still unreliable partly due to the lack of experimentally validated targets [13]. A recent study that compared some published methods for mammalian miRNA targets prediction found that the overlap of identical predictions from the different computational approaches varied between 10% and 50% for a common set of 79 miRNAs [21]. This indicates that false positive predictions could account for a large percentage of all the predicted miRNA target genes and hence need to be filtered by a post-processing step.

Here we present a machine-learning algorithm based on support vector machine (SVM) that can be used as a post-processing software for filtering the targets predicted by other miRNA prediction tools. The prediction system is trained with the experimentally supported animal miRNA targets found in TarBase [13]. Each miRNA:target interaction is mapped into a feature vector in a feature space. The feature space includes various *k*-gram [22] frequencies in the interacting miRNA:target pairs. We use a feature selection procedure to filter out those features with low discriminating abilities, resulting in feature space consisting of 229 features. Support Vector Machines (SVMs) are a class of supervised learning algorithms first introduced by Vapnik [23,24], and have been shown to produce superior results than other supervised learning methods in a wide range of applications. Given a set of labeled training feature vectors (in our case, the positive and the negative miRNA:target interaction pairs), an SVM learns to discriminate between the two classes. The result is a trained model that can be used to classify unlabeled inputs. Our miRNA target prediction system, named MiRTif (miRNA:target interaction filter), achieves sensitivity and specificity of 83.59% and 73.68%, respectively. The prediction system also produces a large AUC of 0.86, which provides a proper measure for the quality of the ranking of a classifier [25]. Higher values of AUC could be interpreted as an indication that positive samples are more likely to receive higher scores from the SVM decision function than negative ones. This is a preferred behavior as it would be possible to rank the query samples according to their likelihood of being true positives. In addition, the majority of the negative miRNA:target interactions (28 of the 38) have been correctly predicted as false ones by MiRTif, meaning the system did not simply classify all input samples as positive, as it might have been expected for a classifier trained with an un-balanced data. The potential problem of over-fitting has also been studied and ruled out by repeating the same training procedure using non-validated, randomly selected targets. We believe that MiRTif can be used effectively as a post-processing filter for miRNA:target interactions predicted by other methods that do not use training sets [10,11,26,27]. With an increasing set of experimentally validated positive and negative target, knowledge-based, machine-learning methods will certainly become more popular. MiRTif can be accessed from .

## Results

### Overall prediction accuracy

The prediction accuracies of the ten-fold cross-validation are listed in Table 1. In the table, TP stands for true positive (correctly predicted positive miRNA:target pairs), FN for false negative (wrongly predicted positive miRNA:target pairs), TN for true negative (correctly predicted negative miRNA:target pairs), and FP for false positive (wrongly predicted negative miRNA:target pairs). The sensitivity, specificity and overall accuracy of the 10-fold cross-validation are 83.59%, 73.68% and 81.97%, respectively. AUC has been shown to be a better measure than accuracy [25], and our system produces a considerably high AUC 0.86.

**Table 1:** Prediction accuracies of the 10-fold cross-validation experiments.

**Positive set**			**Negative set**				
			
***TP***	***FN***	***SE (%)***	***TN***	***FP***	***SP (%)***	***Q (%)***	***AUC***
163	32	83.59	28	10	73.68	81.97	0.86

Prediction accuracies are given in TP (true positive), FN (false negative), TN (true negative), FP (false positive), sensitivity SE = TP/(TP + FN), specificity SP = TN/(TN + FP), overall accuracy Q = (TP + TN)/(TP + FN + TN + FP), and AUC (area under the ROC curve).

Current miRNA target prediction methods are mostly based on similar characteristics of base pairing derived from lin-4 and let-7, and their genetically validated mRNA targets in *C. elegans *[28-32]. Despite the fundamental similarities of those methods, many of their predictions are not in common. In addition, the prediction methods usually produce hundreds of targets for a given miRNA, meaning a large number of them could be false positives. Using the known miRNA:target interaction pairs, our trained SVM classifiers successfully predict most of the false positives: 28 out of the 38 (73.68%) negative samples are correctly predicted to be false interactions. Although this is at the expense of 32 incorrectly predicted positive samples, most of the positive samples (163 out of 195) are correctly predicted.

### Informative features

The feature space used in current SVM classification system includes various *k*-gram frequencies in the interacting miRNA:target pairs (see Methods). Features are ranked according to their ability to discriminate positive and negative samples (the *F *score), and only those with high discriminating abilities are retained for SVM training. These features are hence termed "informative features". Table 2 lists the 25 most informative features, their means, standard deviations and *F *scores. See Methods for the calculation of *F *scores.

**Table 2:** The top 25 most informative features.

**Feature**	***μ***^+^	***σ***^+^	***M***^-^	***σ***^-^	***F***
3-gram, non-seed, mismatch/AU/AU	0.0197	0.0370	0.0000	0.0000	0.5313
2-gram, non-seed, mismatch/AU	0.0526	0.0606	0.0107	0.0353	0.4374
2-gram, entire, mismatch/AU	0.0441	0.0439	0.0160	0.0265	0.3991
3-gram, entire, GC/gap/gap	0.0068	0.0176	0.0228	0.0236	0.3904
3-gram, entire, mismatch/mismatch/gap	0.0060	0.0164	0.0000	0.0000	0.3636
3-gram, non-seed, gap/GU/AU	0.0095	0.0262	0.0000	0.0000	0.3631
3-gram, entire, gap/GU/AU	0.0062	0.0172	0.0000	0.0000	0.3629
3-gram, entire, mismatch/AU/AU	0.0198	0.0312	0.0044	0.0132	0.3457
3-gram, non-seed, mismatch/mismatch/AU	0.0212	0.0422	0.0022	0.0135	0.3417
2-gram, seed, GU/GC	0.0117	0.0352	0.0000	0.0000	0.3337
3-gram, entire, AU/mismatch/GU	0.0054	0.0167	0.0000	0.0000	0.3253
2-gram, entire, gap/gap	0.0838	0.1059	0.1512	0.1030	0.3226
1-gram, entire, GC	0.2399	0.0957	0.2893	0.0678	0.3021
1-gram, non-seed, gap	0.1880	0.1581	0.2841	0.1601	0.3020
2-gram, non-seed, gap/gap	0.1022	0.1406	0.1886	0.1505	0.2969
3-gram, non-seed, GC/mismatch/AU	0.0067	0.0224	0.0000	0.0000	0.2969
1-gram, entire, gap	0.1595	0.1225	0.2273	0.1066	0.2958
3-gram, non-seed, mismatch/mismatch/gap	0.0067	0.0227	0.0000	0.0000	0.2943
3-gram, entire, mismatch/mismatch/AU	0.0135	0.0259	0.0026	0.0111	0.2937
2-gram, entire, GC/gap	0.0199	0.0298	0.0357	0.0243	0.2932
2-gram, entire, GC/GC	0.0630	0.0549	0.0928	0.0471	0.2930
3-gram, entire, GU/GC/gap	0.0002	0.0028	0.0043	0.0115	0.2895
1-gram, non-seed, GC	0.1742	0.1261	0.2377	0.0952	0.2870
3-gram, entire, gap/GU/GC	0.0005	0.0047	0.0056	0.0136	0.2810
3-gram, non-seed, GU/mismatch/AU	0.0064	0.0233	0.0000	0.0000	0.2756

Features are in the format of *k-*gram type, region, and *k-*gram code. For example, "3-gram, non-seed, mismatch/AU/AU" represent a mismatch followed by an AU pair followed by an AU pair in the non-seed region (see Materials and Method – Data representation for the detailed definitions of *k-*gram, region and k-gram code). For each feature, its means and standard deviations in both positive and negative sets are listed. The *F *score is defined as |(*μ*^+ ^- *μ*^-^)/(*σ*^+ ^+ *σ*^-^)|, which measures the discriminating ability of the feature.

It is worth noting that almost all the 25 most informative features in Table 2 come from either the non-seed region or the entire duplex (the only exception is feature "2-gram, seed, GU/GC"). For example, two of the interesting features, the gap frequencies in the non-seed region and the entire region ("1-gram, non-seed, gap" and "1-gram, entire, gap" in Table 2), differ considerably between the positive and the negative samples. We suspect that a highly discriminative *k*-gram code in the non-seed region makes some contribution to the discriminating ability of the same *k*-gram code in the entire region; otherwise we would have observed a high *F *score for this *k*-gram code in the seed region. This observation hints the potential importance of the *k*-gram frequencies in the non-seed region in discriminating positive and negative miRNA:target interaction pairs. In fact, among the 100 most informative features, non-seed region features account for 43, entire region features account for 44, whereas seed region features only account for 13. It again shows that many of the highly informative features lie within the non-seed region.

## Discussion

To study the potential over-fitting problem due to the relatively small number of training samples (195 positives and 38 negatives), we took non-validated targets from miRBase [8] (predicted by miRanda [9]) and randomly partitioned them into sets of 195 and 38. Those targets come from the predictions for the miRNAs listed in Table 3. A total of 7,391 non-validated targets were taken this way. We repeated the random partitioning, i.e., selecting 195 and 38 samples out of the 7,391 non-validated targets, 1,000 times. For each randomly partitioned 195/38 data set, we performed the same feature filtering and SVM parameter optimizing steps. Each random data set was tested by ten-fold cross validation using the optimal SVM parameters. The averaged AUC was 0.66 with a standard deviation of 0.06, a considerable drop comparing with the AUC of 0.86 using the experimentally validated TarBase samples. This result suggests that over-fitting may not be a severe problem otherwise we would have seen equally high AUC values using non-validated targets as the training set.

**Table 3:** List of miRNAs appeared in the training set.

	**MiRNA**
Positive set	let-7, lin-4, lsy-6, miR-273, miR-61, miR-84
	bantam, let-7, miR-1, miR-11, miR-2, miR-278, miR-2a-1, miR-4, miR-7, miR-79
	let-7
	miR-125b, miR-134, miR-181a
	let-7, let-7b, let-7e, miR-1, miR-101, miR-103-1, miR-10a, miR-125a, miR-125b, miR-127, miR-130, miR-132, miR-133, miR-136, miR-141, miR-143, miR-145, miR-15, miR-16, miR-17-5p, miR-196, miR-199b, miR-19a, miR-1b, miR-20, miR-221, miR-222, miR-223, miR-23, miR-23a, miR-24, miR-26, miR-32, miR-34, miR-375, miR-431, miR-433-3p, miR-433-5p, miR-434-3p, miR-434-5p

Negative set	let-7
	mir-276b, mir-278, mir-286, mir-287, mir-288, mir-303, mir-316, mir-317, mir-318
	mir-124, miR-34, mir-375
	let-7b, let-7e, miR-15, miR-16, miR-24, miR-103, miR-141, miR-145, miR-1, miR-19a, miR-34

MiRTif should not be considered as a general tool for miRNA target prediction. It is rather a post-processing filter for the miRNA:target interactions predicted by other seed-sensitive computational methods. Because MiRTif is trained on validated miRNA:target duplexes, both positive and negative training duplexes possess the known miRNA:target binding properties such as the strong seed complementarity and high binding energy. A random duplex (e.g. generated using BLAST) without prescreening by those seed-sensitive programs is not the ideal candidates for using MiRTif.

Machine learning techniques such as Naïve Bayes and SVM have been previously applied to the miRNA target prediction problem [18-20]. Unlike MiRTif, miTarget [20] and NBMiRTar [18] are general target prediction tools (i.e., the targets are predicted from the raw sequences of miRNA and 3'-UTR). The SVM-based miTarget could not easily impose criteria other than features embedded in the encoded duplex. For example, features such as species conservation and the over-representation of conserved adenosines flanking the seed complementary sites [11] are difficult to implement into an SVM feature vector. Being a post-processing filter, on the other hand, MiRTif takes in predicted duplexes from other programs and the abovementioned features are already taken into consideration by these prediction tools. The work by Yan et al. [19] is an ensemble machine learning application that helps to predict miRNA targets. An ensemble algorithm is the one that runs several different algorithms and summarize their outputs to generate the final output. In [19], the ensemble algorithm consists of a Naïve Bayes, a neural network, a decision tree and an SVM algorithm.

The lack of validated negative miRNA:target interaction remains a problem for machine learning-based target prediction approach. For example, the training set of the ensemble algorithm [19] contains only 16 negative samples, in addition to 48 positive ones. Moreover, Sethupathy et al. pointed out that only two out of the 20 or so experimentally refuted miRNA:target interactions for mammals listed in Tarbase are unbiased with respect to various prediction programs [33]. With such a limited number of negative samples, researchers have to rely on artificially generated negative miRNA:target interactions. In NBMiRTar [18], for example, negative miRNA:target interactions were produced by using artificially generated miRNAs and their putative targets predicted by miRanda [9]. The putative targets are believed to be false positive predictions because the query miRNAs are not true. In our study, the potential over-fitting problem due to the lack of sufficient positive and negative samples is addressed by repeating the same training procedure using non-validated, randomly selected targets. As described in Results, the validation for randomly selected target genes suggests that over-fitting may not be a severe problem otherwise we would have seen equally high AUC values using non-validated targets as the training set.

In our study, the validated miRNA:target interactions and the associated binding patterns were retrieved from TarBase, which obtained their information from respective original research papers. Although the duplexes were generated by different prediction tools, many of the duplexes have at least their seed regions experimentally validated. In comparison with miTarget, where the binding patterns were re-computed using RNAfold [34] by the author themselves, we believe that our way of taking samples from TarBase remains to be a better approach for a post-processing miRNA target prediction tool, since otherwise we would have to require all potential users of MiRTif to prepare the duplexes using RNAfold.

One of selection criteria employed by many miRNA target prediction algorithms such as TargetScanS [26], PicTar [10] and miRanda [9] is the perfect or near perfect seed complementarity. As a result, those miRNA:target interactions that are later proven to be negative also have strong seed complementarity as the positive ones do. This explains why most of the highly informative features are within the non-seed region (see Table 2).

The current collection of experimentally validated miRNA:target interaction pairs is still far from being sufficiently comprehensive to give an accurate representation of the target site diversity. Problems with limited dataset can be observed in Table 2, where nine of the 25 features have 0 mean and standard deviation in the negative sets. For example, the 3-gram code "mismatch/AU/AU" in the non-seed region, which ranks number 1 in the list, does not appear in the negative set. Limited training sets may have problems to distinguish highly homogenous compositions, and hence restrict the types of sites that can be predicted. This problem was also pointed out in the TarBase paper [13]. Here we provide the list of miRNAs used for the SVM training (see Table 3). The 195 positive miRNA:target interaction pairs contain 60 miRNAs, while the 38 negative samples contain 24 miRNAs. Since the performance of MiRTif on other miRNAs cannot be tested, Table 3 should provide users a guide about the applicability of MiRTif. With so many miRNA research works going on worldwide, we expect a rapid increase in the experimentally validated target sites. MiRTif, hence, will be updated periodically to provide miRNA target filtering service with an up to date coverage of the validated target sites.

To demonstrate how MiRTif is applied in real world, Table 4 lists a comparison between MiRTif and three *ab initio *target prediction software programs, PicTar, miRBase and TargetScan. Here the example miRNA is hsa-miR-224. This miRNA is the most significantly up-regulated miRNAs in hepatocellular carcinoma patients [35]. The MiRTif discriminant scores were suggested to the experimental biologists working on the miR-224 project along with the predictions made by PicTar, miRBase and TargetScan. Among the predictions, the apoptosis inhibitor-5 (API5) was experimentally validated as a miR-224 specific target [35]. We believe that the value of MiRTif's resides in that it provides an additional suggestion to experimental biologists when choosing targets to validate.

**Table 4:** A comparison between MiRTif and three *ab initio *target predicting software programs, PicTar, miRBase and TargetScan using miR-224, which was discovered to be significantly up-regulated in hepatocellular carcinoma patients.

**Gene**	**Entrez Gene ID**	**Gene description**	**PicTar ranking**	**miRBase ranking**	**TargetScan ranking**	**MiRTif**
H3F3B	3021	H3 histone, family 3B (H3.3B)	1	na	122	+1.69
API5	8539	Apoptosis inhibitor 5	2	na	99	+1.32
ARMCX2	9823	Armadillo repeat containing, X-linked 2	4	198	na	-0.98
CDK9	1025	Cyclin-dependent kinase 9 (CDC2-related kinase)	9	25	na	-0.15
NCOA6	23054	Nuclear receptor coactivator 6	10	204	47	+0.94
ATF2	1386	Activating transcription factor 2	64	19	104	+1.27
NUP153	9972	nucleoporin 153 kDa	168	1	46	-1.46
FOSB	2354	FBJ murine osteosarcoma viral Nncogene homolog B	222	4	125	+0.11

Ranking indicates the rank predicted by respective software program. "NA" indicates that the particular program did not produce an interaction between the corresponding target gene and miR-224. For the discriminant scores under MiRTif, positive scores indicate true predictions while negative scores negative predictions. The score is proportional to the sample's distance from the hyperplane. So a large positive value implies high confidence that the sample lies in the positive class.

## Conclusion

We built an SVM classifier serving as a post-processing filter for miRNA target prediction. The system, MiRTif, takes the targets predicted by other target prediction tools as the input and reports the SVM scores indicting the likelihood of them being true miRNA targets. Tested with the experimentally validated miRNA targets found in TarBase using ten-fold cross validation, the obtained high AUC measurement (0.86) indicates that true positives are indeed ranked higher than false ones. With more biologically validated miRNA:target duplexes in the future, the accuracy increase of knowledge-based, machine-learning approaches such as MiRTif could be anticipated.

## Methods

### Dataset

Known miRNA:target interactions were downloaded from the TarBase version 3.0 [13]. Translationally repressed targets are not separated from cleaved ones. This is because the experimental techniques used to validate targets usually only prove one case but do not disprove others. For example, luciferase reporter assay and immunoblotting assay are widely used to validate translationally repressed targets, but these experiments cannot substantiate whether the given target mRNA is also cleaved by miRNA. Similarly, microarray and real-time RT-PCR assay are often used to validate cleaved targets, but they cannot distinguish translationally repressed targets. Only miRNA:target interactions with reported binding duplexes from drosophila, *C. elegans*, human, mouse, rat and zebrafish were extracted. Duplicated entries and entries with incomplete binding diagrams were removed, resulting in 195 positive and 21 negative miRNA:target duplexes. As the number of negative samples is small, we went on predicting the binding patterns of those known negative interactions without duplexes being reported in the original papers. There are 15 negative miRNA:target interactions from two papers [10,36] that do not have reported binding duplexes. The six experimentally validated negative interactions from Krek's paper [10] have their binding diagrams available from the PicTar website . For the nine negative interactions from the work of Robins [36], we used RNAhybrid [16] to predict the binding duplexes. Two of the 15 negative interactions contain two target sites, as a result, 17 new negative miRNA:target duplexes were added, resulting in a total of 38 negative samples. Both the positive and the negative training sets can be found on the MiRTif website .

### Data representation

**Figure 1:**
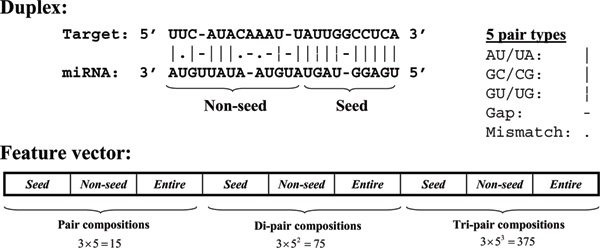
Feature vector encoded for a miRNA:target duplex. Each duplex is partitioned into two parts, with the first part (the seed) covers nucleotide 1 to 9 from the 5' end of the miRNA, and the second part (the non-seed) covers the rest of the duplex. Five types of base-pairing are defined. A total of 465 features, consisting of the 1-gram, 2-gram and 3-gram frequencies of the five pairing types, are encoded into a vector representing a miRNA:target duplex.

For a given miRNA:target duplex, the gaps at both ends are firstly removed, if any. The duplex is then partitioned into two parts, with the first part covers nucleotides 1–9 from the 5' end of the miRNA, and the second part covers the rest of the duplex. These two parts are named "seed" and "non-seed" regions, respectively (see Figure 1). We define five types of pairing in a duplex: AU (AU or UA pair), GC (GC or CG pair), GU (GU or UG pair), gap and mismatch. A *k-*gram is known as a subsequence of length k. For the case of miRNA:target duplex, we define a *k-*gram as a duplex fragment of length *k*. Therefore, a 1-gram contains 5 different pairing types, which we call them "1-gram codes". Similarly, 2-gram and 3-gram contains 5^2 ^and 5^3 ^codes, respectively. For each miRNA:target interaction, we compute the 1-gram, 2-gram and 3-gram frequencies in the seed, non-seed and entire duplex, giving 3 × 5 + 3 × 5^2 ^+ 3 × 5^3 ^= 15 + 75 + 375 = 465 features. The 2-gram and 3-gram codes are counted from the 5' end of the miRNAs. For example, in Figure 1, there are two but not one occurrences of AU/GC (AU pair followed by GC pair) in the seed region, because the 2-gram code is counted from 5' of the miRNA.

### Feature selection

Although state-of-the-art classifiers such as neural networks and support vector machines can accommodate redundant and noisy features, removing non-informative features may still improve the performance of these classifiers [37]. Therefore, a feature selection procedure adopted from Golub et al. [38] was performed. For each feature *x*_*j*_, *j *= 1,..., 465, the mean $\mu_{j}^{+}$($\mu_{j}^{-}$) and the standard deviation $\sigma_{j}^{+}$($\sigma_{j}^{-}$) for both the positive and the negative sets are calculated. The *F *score is defined as $F(x_{j})=\left|\frac{\mu_{j}^{+}-\mu_{j}^{-}}{\sigma_{j}^{+}+\sigma_{j}^{-}}\right|$, which can be used to rank the features according to how well they discriminate the positive and negative samples. In this work, we used a threshold of 0.1 for feature selection, that is, if a feature has an *F *score greater than 0.1, it is retained; otherwise it is removed from the feature vector.

The F-value threshold was chosen based on a survey of the number of retained features using various F-value cut-off values. Using a cut-off value of 0.05, 0.06, 0.07, 0.08, 0.09, 0.10, 0.20, 0.30, 0.40 and 0.50, the numbers of retained features are 327, 312, 290, 255, 242, 227, 82, 19, 2, and 1, respectively. Although we would like to remove the redundant and irrelevant features, using an F-score cut-off greater than 0.20 would reduce the number of features drastically, down to 82 features from 227. Hence we decided to use 0.1 as the F-value threshold.

The final feature vector contains 229 features with *F *scores greater than 0.1. These 229 informative feature can be obtained from the MiRTif website .

### Support vector machines

Support Vector Machine (SVM) was first introduced by Vapnik [23,24]. It has been used extensively in a wide range of areas of classification and regression and has shown excellent empirical performance. In bioinformatics investigation, SVM has been utilized to study problems such as microarray gene expression data analysis [39], protein secondary structure prediction [40], prediction of protein-protein interaction [41] and RNA-protein interaction [42], prediction of protein subcellular localization [43-45], protein remote homology detection [46-49], and classification of real and pseudo miRNAs [50,51]. SVM is a learning algorithm that, upon training with a set of positively and negatively labeled samples, produces a classifier that can then be used to identify the correct label of unlabeled samples. Each sample is described by a feature vector, which is often not linearly separable. SVM thus maps the input vectors into a high dimensional space *H *and construct an optimal hyperplane that divides the positive and the negative samples with the maximum margin of separation between the hyperplane and the closest points from both classes.

The SVM algorithm requires the solving of a quadratic optimization problem. To simplify the problem, SVM does not explicitly map the feature vectors of all the samples to the space *H*. Instead, mapping is done implicitly by defining a kernel function $K(\vec{x},\vec{y})$ between two samples with feature vectors $\vec{x}$ and $\vec{y}$ as $K(\vec{x},\vec{y})=\varphi (\vec{x})\cdot \varphi (\vec{y})$, where *ϕ *is the mapping to the space *H*. A detailed description of the mathematics behind SVM can be found in an article by Burges [52]. In the present study, we used soft-margin SVM implementation in SVM^light ^(version 6.01) created by Joachims [53]. The package can be downloaded from  and is free for academic use.

### Hyper-parameter selection

The training of SVM requires selection of several hyper-parameters, whose values determine the function that SVM optimizes and hence have a crucial impact on the performance of the trained SVM classifiers. In this work, the optimal hyper-parameter set was selected by a 10-fold cross-validation on the entire dataset. This approach has been shown to be a robust method for hyper-parameter tuning [54]. We chose the widely used radial basis function (RBF) kernel that is defined as $K(\vec{x},\vec{y})=e^{-\gamma {\left\|\vec{x}-\vec{y}\right\|}^{2}}$, *γ *> 0. A grid search over the values of the parameters *c*, *j *and *γ *was performed. Parameter *c *controls the trade-off between training error and margin, *j *is the cost factor by which training errors on positive samples (false negatives) outweigh errors on negative samples (false positives), and *γ *controls width of the RBF kernel. For each parameter combination, we measured AUC after 10-fold cross-validation. A receiver operating characteristic (ROC) curve is a plot of true positives as a function of false negatives [55]. An AUC of 1 means perfect separation of positive examples from negative ones; whereas an AUC of 0.5 indicates random separation. AUC was chosen as the performance measure because it is integrated over all threshold values. The best cross-validation performance was obtained with *c *= 300, *j *= 0.14 and *γ *= 0.1. The performance of the final SVM classification is also measured by the quantity of true positives (*TP*), true negatives (*TN*), false positives (*FP*), false negatives (*FN*), sensitivity (*SE*), specificity (*SP*) and overall accuracy *Q*. The definition of sensitivity, specificity and overall accuracy are listed below:

$$SE=\frac{TP}{TP+FN}$$

$$SP=\frac{TN}{TN+FP}$$

$$Q=\frac{TP+TN}{TP+TN+FP+FN}.$$

## Competing interests

The authors declare that they have no competing interests.

## Authors' contributions

YY collected the raw data, designed and implemented the cross validation experiments. YPW and YY implemented the web server. YY, YPW and KBL were all involved in analyzing the validation results. YY and KBL prepared the manuscript. KBL initialized and supervised the whole project. All authors read and approved the final manuscript.
